# Dysregulation of Ion Channels and Transporters and Blood-Brain Barrier Dysfunction in Alzheimer’s Disease and Vascular Dementia

**DOI:** 10.14336/AD.2023.1201

**Published:** 2024-08-01

**Authors:** Ruijia Liu, Jenelle M Collier, Nana-Hawwa Abdul-Rahman, Okan Capuk, Zhongling Zhang, Gulnaz Begum

**Affiliations:** ^1^Department of Neurology, The First Affiliated Hospital of Harbin Medical University, Harbin, Heilongjiang, China.; ^2^Department of Neurology, The Pittsburgh Institute for Neurodegenerative Diseases, University of Pittsburgh, Pittsburgh, PA, USA.; ^3^Center for Neuroscience, University of Pittsburgh, Pittsburgh, PA, USA.; ^4^University of Pittsburgh School of Medicine, Pittsburgh, PA, USA.

**Keywords:** Alzheimer’s disease, Blood-brain barrier, Ion channels, Ion transporters, Vascular dementia

## Abstract

The blood-brain barrier (BBB) plays a critical role in maintaining ion and fluid homeostasis, essential for brain metabolism and neuronal function. Regulation of nutrient, water, and ion transport across the BBB is tightly controlled by specialized ion transporters and channels located within its unique cellular components. These dynamic transport processes not only influence the BBB’s structure but also impact vital signaling mechanisms, essential for its optimal function. Disruption in ion, pH, and fluid balance at the BBB is associated with brain pathology and has been implicated in various neurological conditions, including stroke, epilepsy, trauma, and neurodegenerative diseases such as Alzheimer’s disease (AD). However, knowledge gaps exist regarding the impact of ion transport dysregulation on BBB function in neurodegenerative dementias. Several factors contribute to this gap: the complex nature of these conditions, historical research focus on neuronal mechanisms and technical challenges in studying the ion transport mechanisms in *in vivo* models and the lack of efficient *in vitro* BBB dementia models. This review provides an overview of current research on the roles of ion transporters and channels at the BBB and poses specific research questions: 1) How are the expression and activity of key ion transporters altered in AD and vascular dementia (VaD); 2) Do these changes contribute to BBB dysfunction and disease progression; and 3) Can restoring ion transport function mitigate BBB dysfunction and improve clinical outcomes. Addressing these gaps will provide a greater insight into the vascular pathology of neurodegenerative disorders.

## 1. Introduction

The blood brain barrier (BBB) is a highly specialized physical and biochemical barrier that regulates the exchange of substances between the brain and the peripheral circulation [1-3]. Its restrictive and selective permeability to substances prevents rapid ionic or metabolic changes within the brain [4]. Unlike a single physical structure, the BBB is a result of functional association between endothelial cells (ECs), basal lamina, pericytes, and astrocytic perivascular endfeet that serves to maintain the structural integrity and ion homeostasis within the brain [1, 4].

**Figure 1.**
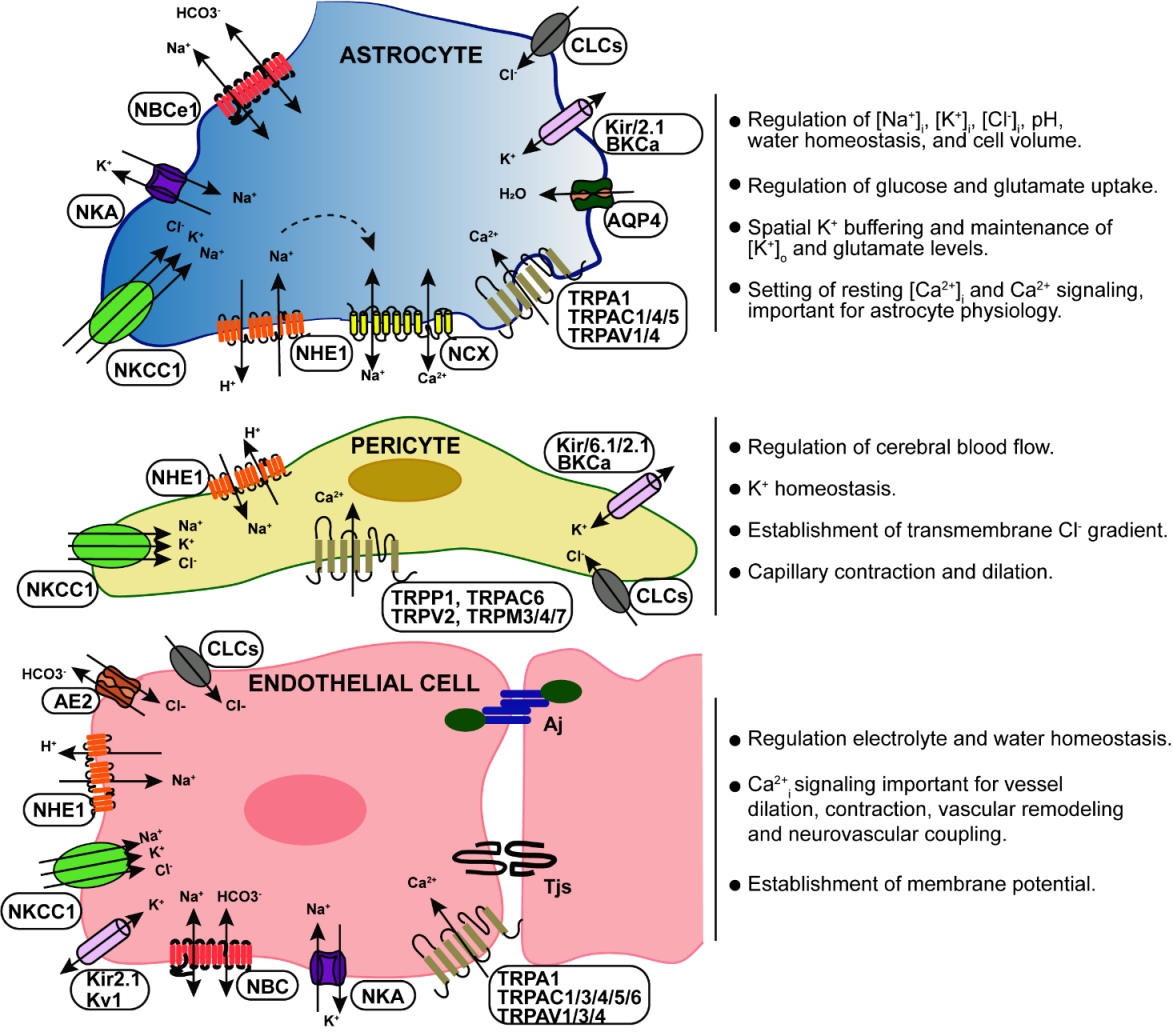
**Key blood-brain barrier ion transport systems**. Ion transporters and channels expressed within the BBB mediates the movement of Na^+^, K^+^, Cl^-^, HCO_3_^-^, H^+^ and Ca^2+^ into and out of the BBB specific cells. The BBB ion transport is important for regulation of key ionic concentrations in the brain (H^+^, K^+^, Ca^2+^), the uptake and extrusion of trace metals, fluid secretion, intracellular volume, pH and Ca^2+^ homeostasis.

The BBB plays a crucial role in the sustained regulation of ionic composition in the brain’s interstitial fluid [4, 5]. For example, Na^+^, K^+^, Ca^2+^, Cl^-^, and HCO_3_^-^ are the major ions in the central nervous system (CNS), which should be kept at an optimal level for neural and synaptic signaling functions [6, 7]. These ions exhibit an asymmetric distribution between luminal and abluminal membranes, with plasma concentrations of Na^+^ (148-155); K^+^ (3.9-4.6); Cl^-^ (113-114); Ca^2+^ (2.5), and cerebrospinal fluid (CSF) Na^+^ (152-156); K^+^ (2.99-3); Cl^-^ (126-129); Ca^2+^ (1.2-2.0) at mmol/ liter of H_2_O [4]. The transport of these ions across the BBB is tightly regulated by the function of ion channels and transporters within the BBB [6, 7] (Table 1). Importantly, ECs, pericytes, smooth muscle cells and astrocytes express different transporters, receptors, active efflux pumps, ion channels and regulatory molecules. This multifaceted expression allows for the selective transport of ions across the BBB, thereby maintaining ionic balance in the brain [8-10] (Fig. 1). Disruption of ionic gradients at the cellular level can have significant effects on brain function, potentially leading to damage or dysfunction [6, 7, 11].

Many cerebrovascular pathologies are closely associated with BBB dysfunction, where changes at the BBB can lead to or enhance disease development [12, 13]. Emerging new studies indicate that dysfunction in ion transport at the BBB is not only associated with acute brain injuries but also plays a role in various neurological disorders, including stroke, epilepsy, multiple sclerosis, vascular dementia (VaD), and Alzheimer’s disease (AD) [14-16]. Therefore, understanding the mechanisms behind ion transport dysregulation at the BBB is crucial for the development of new therapeutic strategies aimed at addressing various brain diseases. In this review, we present the current knowledge concerning ion transporters and channels expressed by the BBB specific perivascular cells, including their molecular composition, and function in the context of neurological disorders. We also shed light on unanswered questions related to ion transport dysregulation as potential therapeutic targets for neurodegenerative diseases.

**Table 1:** Key ion channels and transporters within BBB and their function.

Ion channel/Transporter	Transported ion	Localization			Main function at BBB
		ECs	Astrocytes	Pericyte	
**Na^+^-K^+^-ATPase (NKA)**	Na^+^, K^+^	+	+	+	K^+^ transport, maintenance of [Na^+^] gradient at the BBB, H_2_O homeostasis; Regulation of glycolysis and glutamate uptake
**K^+^ channels**	K^+^	+	+	+	Regulation of membrane potential, K^+^ buffering, CBF and neurovascular coupling
**Chloride channels**	Cl^-^	+	+	?	Cl^-^ transport; cell volume regulation
**TRP Channels**	Ca^2+^	+	+	+	Regulation of Ca^2+^ influx, vascular tone, and CBF
**NHE1**	Na^+^, H^+^	+	+	?	Removal of intracellular H^+^; pH regulation
**NBC**	Na^+^, HCO_3_^-^	+	+	?	pH and HCO_3_^-^ regulation
**NKCC1**	Na^+^, K^+^, Cl^-^	+	+	?	Regulation of Cl^-^, and K^+^, homeostasis; H_2_O transport
**NCX**	Na^+^, Ca^2+^	+	+	?	Mediates Ca^2+^ efflux and maintains cellular Ca^2+^ homeostasis
**AQP4**	H_2_O	-	+	-	Regulation of H_2_O homeostasis
**AE2**	Cl^-^, HCO_3_^-^	+	?	?	Acid loaders; pH regulation

## 2. Overview of the BBB structure and functions

The BBB is formed by a monolayer of tightly sealed microvascular ECs that constitute the walls of the vessels. The abluminal side of the endothelium is covered by a 30-40 nm thick basal lamina, mural cells (such as vascular smooth muscle cells and pericytes), and astrocytic endfeet [1, 2]. Additionally, perivascular macrophages reside between the vascular cells and astrocytic endfeet, providing immune surveillance [2]. ECs are the central elements of the microvasculature that form the BBB. They are characterized by a flattened structure, expression of inter endothelial tight junctions (TJs), and the presence of very few caveolae at the luminal surface [1]. These features contribute to the maintenance of integrity, barrier function, and cellular communication at the BBB. The paracellular transport of substances from blood across the endothelium is regulated by the junctional complexes including TJs, adherens junctions (AJs), and gap junctions that holds the ECs together [2, 17]. The junctional complexes are highly interdependent in regulating EC permeability. The TJs are composed of transmembrane proteins claudin (claudin-1, -3, -5, and -12), occludin, junctional adhesion molecules (A, B, and C) and membrane-associated guanylate kinase protein family zonula occludens (ZO-1, -2, and -3) [18, 19].

The TJs are important to regulate the permeability of the BBB via sealing the paracellular space, preventing diffusion of molecules between the ECs. The claudins, specifically CLDN-5, are the key structural components of the TJs, that contributes to the reduced paracellular ion movement [20]. Mice deficient in CLDN-5 exhibit a selective disruption of the BBB, and increased permeation of tracers less than 1 kDa [21]. Occludin is also present in the filaments of TJs and helps regulate adhesion properties between cells as well as interacting with the inner cellular scaffolding proteins and the actin cytoskeleton [22]. Studies have indicated that downregulation of CLDN-5 and occludin results in decreased barrier tightness and enhanced monocyte migration across the BBB [23]. Loss of CLDN-5 and occludin in the cerebral cortex is found to be associated with synaptic degeneration in post-mortem brains of AD patients [24]. ZOs interact with claudins, occludin, and JAMs to anchor the membrane proteins, tethering them to the actin cytoskeleton [25]. Severe decreases in retinal vascular ZO-1 correlating with increased BBB permeability and abundant arteriolar Aβ40 deposition were identified in AD patients [26].

AJs are intercellular connections that facilitate cell-cell communication and stabilize EC contacts, regulating permeability for large plasma components [19]. They include transmembrane proteins like cadherins, responsible for the adhesion and catenin’s, which support cadherin association and signaling regulation across the BBB. In brain ECs, the primary transmembrane AJ protein is Ve-cadherin, working with catenin, plays an important role in the maintenance of cell-cell adhesion, contact inhibition, cytoskeleton remodeling, and intercellular signaling [22]. The intricate interactions between these transmembrane proteins create a molecular architecture that maintains BBB integrity under mechanical stress. Loss of the AJs can lead to a loss of BBB integrity and its increased permeability seen in various CNS disorders [27].

ECs express transport proteins that facilitate the transport of selective substances, regulate nutrient delivery, and waste removal from brain parenchyma. These transporters use passive and active transport to regulate processes such as signal transduction, endocytosis, transcytosis, and molecular transport critical for transport of nutrients and waste materials within the brain [28]. ECs are equipped with ion transporters/channels (Fig. 1) such as sodium pumps, calcium channels, acid exchangers, proton/bicarbonate transporters and potassium channels. These are critical in regulating the electrophysiological activity of neuronal cells, and maintaining the sodium concentration gradient at the BBB that drives sodium dependent transport processes [29], which are the focus of this review.

Mural cells cover the abluminal surface of the blood vessels and can be classified into two major categories: vascular smooth muscle cells (VSMC) and pericytes. VSMCs are predominantly located in large diameter vessels including arteries, arterioles, and veins, where they contribute to regulation of blood flow [30]. On the other hand, pericytes are typically associated with small diameter vessels, and capillaries, partially covering the endothelium and extending their contacts across hundreds of micrometers of capillary length [31]. Due to their close proximity to ECs, pericytes play a crucial role in regulation of blood flow by controlling the diameter of capillaries through the release of vasoactive substances [32]. Single cell transcriptomics studies have revealed that capillary pericytes express a diverse array of ion channels and G protein coupled receptors, which are involved in mediating the contraction and dilation response to vasoactive mediators [33].

Lastly, astrocytes play a crucial role in the formation and maintenance of the BBB. Astrocytes are the primary contributors to establishing, upkeeping, and repairing the BBB [34, 35]. An important feature of astrocytic interaction with the BBB are the perivascular endfeet that encompass the abluminal surface of ECs and the surrounding mural cells, creating a continuous interface between glial and vascular components. Astrocytic endfeet are highly enriched with ion channels/ transporters, glucose transporters and G-protein coupled receptors [36]. Through these astrocytes dynamically mediate many regulatory functions, including maintenance of BBB integrity, mechanical and metabolic support, endothelial transport, innate immune response, and cerebrovascular regulation [35-37]. Further, astrocytic endfeet processes also secrete basement membrane proteins such as laminins which contribute to stabilizing the BBB [38]. Astrocytes are the major secretors of sonic hedgehog (Shh) Wnt’s, and norrin proteins that were identified as crucial factors of BBB maintenance [39].

Among the BBB ion transporters and channels (Figure 1), the Na^+^/K^+^ ATPase mediates the active transport of Na^+^ and K^+^ across the BBB that is crucial for maintaining brain water and electrolyte homeostasis [4]. Multiple secondary active transporters such as Na-K-Cl co-transporter, Na/H exchanger, Na-HCO3 co-transporter and Na/Ca exchangers, alongside a variety of ion channels including inward rectifier Kir channels, ATP-sensitive K, and Na channels, function collectively to regulate intracellular ion concentrations, cellular volume, and pH [4, 16]. These transporters and channels play a pivotal role in the vectorial movement across the BBB, maintaining the precise concentrations of Na^+^, K^+^, Cl^-^, and HCO_3_^-^ in the brain’s extracellular fluid, critical for regulating the electrophysiological activity of neurons. Further, selective expression of channels such as Kir4.1 and aquaporin 4 (AQP4) at the endfeet polarize astrocytes and facilitate the directional flow of K^+^ and water molecules to maintain ion homeostasis and EC volume [40]. This orchestrated regulation contributes significantly to maintaining the brain's microenvironment and facilitates vital processes such as nutrient transport while preventing the entry of harmful substances into the brain.

## 3. BBB dysfunctions in AD and VaD

Disruption/dysfunction of the BBB is considered a hallmark associated with several brain pathologies including neurodegenerative diseases. A large body of evidence indicate that damage to BBB is a characteristic feature of AD, occurring early in the disease process, even before the onset of dementia and neurodegeneration [41, 42]. Brain capillary leakages and perivascular accumulation of blood-derived fibrinogen, thrombin, albumin, and immunoglobulin G (IgG), pericyte and EC degeneration, loss of BBB TJs, and red blood cell extravasation, are some of the features of BBB damage implicated in AD [43].

Activation of inflammatory and oxidative stress signaling pathways are the primary events that cause BBB disruption. Cytokines, amyloid β (Aβ) and tau-proteins are the stimulus of inflammation and increase in proinflammatory mediators and reactive oxygen species (ROS)/reactive nitrogen species (RNS) in ECs, astrocytes and pericytes are the major drivers of BBB damage [44]. At cellular level, degeneration of ECs associated with reduction in EC thickness, length, and density, loss/ damage to TJ proteins, has been detected in AD brains [45]. In addition, pericyte degeneration due to the toxic effects of Aβ also contributes to EC degeneration in AD [46, 47] and facilitates extravasation of toxic plasma proteins into brain parenchyma.

BBB breakdown is also most pronounced in individuals carrying APOE*e4 allele which is shown to be associated with microvascular injury and pericyte degeneration, but the molecular mechanisms are not clear [48, 49]. Increased expression of proinflammatory mediators such as cyclophilin A and matrix metalloproteinase 9 (MMP-9) that are known to degrade BBB TJ proteins are reported to be overexpressed in AD [47]. Changes in the morphology of astrocytes in AD brain have also been shown to cause BBB breakdown [50]. The depolarization of astrocyte endfeet weakens the integrity of BBB, which was reported in the Tg-ArcSwe mouse model of AD [51]. Decreased vascular coverage and loss of AQP4 expression by the endfeet in AD patients and animal models has been shown to be associated with Aβ-accumulation and BBB damage [52, 53]. The perturbed AQP4 expression in the astrocytic end-feet has also been observed to cause inflammation and increase in neurofibrillary tangles in the human AD brain [54].

VaD refers to the loss of cognitive function because of reduced cerebral blood flow (CBF) to the brain, largely caused by damage to the cerebral blood vessels. AD and VaD together account for >70% dementia cases and share similar vascular pathologies and cognitive decline [55]. BBB disruption/dysfunction is considered as a core mechanism in VaD contributing to disease pathology and progression [56]. Loss of EC integrity, degeneration of pericytes, astrocyte endfeet swelling and retraction from the vessel wall, loss of TJs, and brain capillary leakages are the known factors disrupting the BBB integrity subsequently leading brain hypoperfusion [57, 58]. Studies have indicated that oxidative stress, hypoxia, and inflammation are the major drivers of BBB dysfunction and cerebral hypoperfusion. Hypoxia upregulates oxidative stress to produce NO, ROS and free radicals that damage the BBB ECs leading to reduced CBF [59, 60]. Vascular hypoxia causes increased secretion of inflammatory molecules including soluble LRP1, cyclophilin A, MMPs (MMP-2 and MMP-9), IL-1, IL-6, TNF-a and TLR4 that cause damage to the ECs, astrocytes, and surrounding neurons to enhance BBB permeability [61].

In a rat model of VaD (bilateral common carotid artery stenosis (BCAS)), reactive astrocyte-derived lipocalin-2 (Lcn2) in the hippocampus has been shown to mediate BBB damage leading to neuroinflammation and cognitive impairment [62]. Lcn2-deficient mice showed less BBB permeability, cognitive decline, neuronal loss, glial activation, and cytokine production [62]. BBB disruption due to degeneration of pericytes causes disruption in white matter circulation, deposition of fibrinogen, and reduction of CBF that induces further damage to the myelin, axons and oligodendrocytes [63]. Recent studies have shown that, animals subjected to chronic cerebral hypoperfusion upregulates intercellular adhesion molecule 1 (ICAM-1) and vascular adhesion molecule 1 (VCAM-1) in the vascular ECs that was associated with cognitive impairment [64].

Together, insights from the literature underscore the significance of BBB dysfunction as a critical factor in neurodegenerative disorders such as AD and VaD. Restoration of BBB function has emerged as a promising therapeutic target to effectively impede the progression of neurodegenerative mechanisms and alleviate the associated clinical deficits.

## 4. Key ion transporters and channels at the BBB

### 4.1. Na^+^/K^+^-ATPase (NKA)

The NKA is the main transporter that couples metabolic energy to ion transport at the BBB and is highly expressed by the ECs and astrocytes ensheathing the BBB at the abluminal side [4, 29]. NKA is crucial for maintaining optimal ion levels, particularly a high Na^+^ concentration and low K^+^ level in the brain interstitial fluid. This balance is crucial for establishing the Na^+^ concentration gradient at the BBB, facilitating essential Na^+^ dependent transport processes important for brain water and electrolyte homeostasis [4, 65]. Three different α subunits and two β subunits of NKA have been reported to be expressed at the BBB and within ECs, it is primarily located in the abluminal membranes [4, 5]. However low NKA activity has also been reported in luminal membranes suggesting the presence of different isoforms on these two surfaces [4]. Dysfunction in distinct NKA isoforms plays a crucial role in neurodegenerative diseases; deficiency in α1/α3 subunits accelerates neuronal loss and diminishes pump function. Conversely, the overactivation of the α2 subunit contributes to neuronal loss in AD [66].

In astrocytes, NKA constitutes the major pathway of Na^+^ efflux, however it is also known to play critical role in the regulation of extracellular K^+^ concentration ([K^+^]o) [67]. In a healthy brain, astrocytic NKA activity is required for increased glucose uptake, breakdown of glycogen, stimulation of glycolysis as well as the production of lactate [68]. However, impaired NKA function in astrocytes negatively impacts glutamate clearance, leading to abnormally high neuronal activity affecting memory functions in patients [69, 70]. Reduced NKA activity can also cause increase in intracellular calcium concentration ([Ca^2+^]_i_), due to the reverse mode activation of Na^+^/Ca^2+^ exchanger (NCX), leading to excitotoxicity [67]. Altered NKA activity in the perivascular astrocytes may disrupt glutamate transport pathways [71], potentially disrupting ionic homeostasis crucial for BBB function, and potentially contributing to the development of neurodegenerative disorders.

Impaired NKA expression and activity have been associated with acute brain injuries and chronic neurodegenerative disorders. In ischemic stroke, reduced NKA activity in neurons and astrocytes leads to accumulation of intracellular sodium (Na^+^_i_) that further triggers Na-K-Cl cotransport and Na^+^/H^+^ exchanger 1 (NHE1) activation, increasing Na^+^ influx. With compromised NKA function unable to balance these transport processes, Na^+^_i_ accumulation worsens, causing EC swelling [16], damaging BBB integrity and leading to brain edema in stroke brains [72]. Similarly, persistent decrease in NKA activity is observed in various traumatic brain injury (TBI) models, contributing to BBB damage [73, 74]. Dysfunctional NKA activity and intracellular Na^+^ accumulation has been associated with neuroaxonal loss in multiple sclerosis patients [75], sustained membrane depolarization and NMDA receptor overactivation and excitotoxicity in Huntington’s disease (HD) [76]. Additionally, significantly decreased NKA activity has been observed in motor neurons of the (SOD1) G93A mouse model of Amyotrophic lateral sclerosis [77]. However, further studies are needed to precisely determine the impact of dysfunctional NKA activity on BBB function.

### 4.2. Ca^2+^ channels/transporters

Ca^2+^ channels and their downstream signaling mechanisms play critical roles in mediating the integrity of the BBB and contribute significantly to CBF regulation [78, 79]. ECs express many different types of Ca^2+^ channels, including Ca^2+^ transporters like the NCX, Ca^2+^ ATPase, P2X receptors, L-Type Ca^2+^ channels important for the regulation of BBB integrity and CBF [29, 80]. Transient receptor potential (TRP) channels are another important class of Ca^2+^ influx channels expressed abundantly by ECs and astrocytes [81, 82]. Among the 7 different subfamilies, the TRPC, TRPV, TRPM, and TRPA1 are known to modulate BBB function. TRPC channels are the best characterized and have been proposed to mediate EC [Ca^2+^]_i_ which is a crucial second messenger that leads to various vascular responses, including changes in vascular tone, alteration in BBB permeability, vascular remodeling and oxidative damage [83]. TRPC6 is mainly activated by diacylglycerol, mechanical stretch, and exposure to ROS to cause Ca^2+^ influx that facilitates increases in EC permeability [84]. TRPC3 overexpression/hyperactivation in ECs has been associated with increased BBB permeability and vasogenic edema observed in response to status epilepticus in mice [85]. Activation of TRPA1 within capillary ECs, contribute to Ca^2+^ mediated dilation of arterioles and increased blood flow in the somatosensory cortex of the mouse brain, indicating its important role in mediating neurovascular coupling [86].

Astrocytes express TRPA1, TRPC1/4/5, and TRPV4 channels (reviewed in [82]). The TRPA1 channels in hippocampal astrocytes mediate spotty Ca^2+^ transients that contribute to resting [Ca^2+^]_i_ [87]. The TRPC1/4and 5 channels provide a pathway for store operated Ca^2+^ entry (SOCE) and contribute to modulating cytosolic Ca^2+^ signals and generating a substantial Na^+^ entry [82]. TRPV1 in astrocytes are preferentially localized in the endfeet and its activation trigger expression of early gene, c-Fos involved in cell proliferation and differentiation [88]. TRPV4, also expressed in astrocytic endfeet are activated in response to hypo-osmotic stress and cell swelling, stimulate Ca^2+^ induced Ca^2+^ release and amplify neurovascular coupling response [89]. Under pathological states, astrocytic TRP channels can be activated by ROS, RNS, proinflammatory factors, and pathological markers of neurodegenerative diseases, such as Aβ, which disrupts the Ca^2+^ homeostasis [82]. Astrocytic Ca^2+^ overload can cause excessive activation of astrocytes that can cause BBB disruption by releasing pro-inflammatory factors. Capillary pericytes express 11 different isoforms of TRP channels (TRPC1/3/4/6, TRPM3/4/7, TRPML1, TRPP1, TRPP3 and TRPV2). Among these isoforms, members of TRPC subfamily and TRPM7 play essential roles in mediating SOCE intracellular Ca^2+^ signaling in pericytes. Pericytes exhibit sensitivity to a range of mechanical perturbations, and TRPP1, TRPV2, TRPC1/6 and TRPM4 channels are implicated in mechanosensation- evoked Ca^2+^ entry influencing the vascular tone (reviewed extensively in [33]).

Abnormal Ca^2+^ signaling due to dysfunctional Ca^2+^ channels and transporters has been studied extensively in ischemic stroke models. Studies using nifedipine, (Ca^2+^ channel blocker), Ca^2+^ chelators, or inhibition of protein kinase C (PKC) have shown decreased Ca^2+^_i_ levels, reducing EC permeability and BBB damage in cultured ECs and *ex vivo* brain slices during ischemic conditions [90-92]. Dysfunctional TRP channels, particularly TRPM2 and TRPM4, have been implicated in ischemic stroke. Inhibition of TRPM2 preserved EC integrity, reduced brain edema and preserved TJs [93], while pharmacological inhibition of TRPM4 mitigated oncotic cell death [94] and decreased BBB permeability and improved cerebral perfusion [95]. The upregulation of sulfonylurea receptor 1 (SUR1)-TRPM4 channel has been seen in microvascular ECs, astrocytes and pericytes in both humans with stroke and rat models of ischemic stroke [96-98]. Activation of SUR1-TRPM4 channels is shown to mediate PAR-1 and Ca^2+^ dependent secretion of MMP9 by the brain ECs, causing BBB damage [99]. Pharmacological blockade of SUR1 channel decreased MMP9 secretion, mitigated BBB damage, and reduced hemorrhagic transformation in stroke models [99-102].

The involvement of Ca^2+^ channels in neurodegenerative disorders has been extensively documented [103]. In mouse models, the application of neurotoxins mimicking Parkinson’s disease (PD) leads to the downregulation of TRPC1 and store operated Ca^2+^ influx resulting in the death of dopaminergic neurons. Conversely, the activation or overexpression of TRPC1 protects against neurotoxin mediated cytotoxicity [104]. TRPC1 acting as a potential store operated Ca^2+^ channel is upregulated in response to mutant huntingtin protein expression in mouse model of HD, inducing neuronal damage. Inhibition of store operated Ca^2+^ entry or knockdown of TRPC1 downstream regulator STIM2 provides neuroprotection and rescues dendritic spine deficiency [105].

### 4.3. K^+^ channels

K^+^ channels, such as inwardly rectifying potassium channels (Kir), two-pore domain K^+^ channels (K2P), KCa channels and voltage-gated K^+^ channels (Kv) are known to play important roles in the regulation of BBB permeability and control of CBF [106]. The inward rectifier Kir2.1 channel is the primary K^+^ channel type in brain capillary ECs. Along with voltage-gated potassium channel Kv1, it contributes to the establishment of membrane potential and mediates K^+^ efflux into the brain interstitial fluid [107]. Recent findings highlight that EC Kir2.1 are amplifiers of retrograde electrical signaling in the cerebral vasculature, which is critical for K^+^ evoked increases in CBF [107, 108]. Action potential induced extracellular K^+^ released from perivascular neurons and astrocytes activates Kir2.1 in ECs, resulting in a rapidly propagating endothelial hyperpolarization, vessel dilation and a subsequent increase in local CBF [107, 109].

Capillary pericytes express extremely high levels of Kir6.1 and, to a lesser extent, Kir2.2 and Kir2.1 channels [33]. The Kir6.1 and SUR 2 subunits constitute the vascular form of ATP sensitive potassium channels (KATP) channels, which play a major role in coupling membrane hyperpolarization to CBF control [33, 110]. KATP channels in capillary pericytes can be modulated by glucose concentration, adenosine, and endothelin-1 (ET-1). A decrease in cellular glucose activates KATP channels due to lower ATP:ADP ratio, which causes pericyte hyperpolarization and transmits the signal to capillary ECs, increasing blood flow via a Kir2.1 channel-dependent mechanism [111]. Endogenous adenosine from neurons and glial cells can also stimulate KATP channels through the A2AR-Gαs-AC-PKA signaling cascade to conduct electrical response, which enhances CBF [112]. However, ET-1 would inhibit KATP channels to prevent hyperpolarization and dilation of pericytes, even diminish gap junctions between pericytes [31]. The role of Kir2.2 in pericytes remains poor explored, but it was predicted to propagate hyperpolarizing signals from capillary pericytes to upstream vessels [33]. Recently, RNA sequencing transcriptomic analysis of brain vascular pericytes indicated that Kir4.1 functionally expressed in pericytes are engaged in maintenance of K^+^ homeostasis [113]. Further studies are needed to explore the role of K^+^ channels in pericytes.

In astrocytes, four types of K^+^ channels including Kir4.1, K2P channels, large-conductance KCa (BK) and Kv channels have been identified, of which Kir4.1 and BK are abundantly expressed in endfeet [106]. Kir4.1 plays a pivotal role in “K^+^ buffering”, translocating excess extracellular K^+^ into the capillaries, and maintains water/K^+^ homeostasis together with AQP4 in astrocyte endfeet [114]. In addition, BK channels were found co-localized with AQP4 in perivascular astrocytic endfeet of rat hippocampus and cerebellum, suggesting the involvement of BK channels in K^+^ redistribution and regulation of CBF [106]. Astrocytic Ca^2+^ waves triggered by neuronal activity can activate BK channels directly at the endfeet, resulting in extracellular K^+^ accumulation and rapidly induce both vasodilation and vasoconstriction [80, 115].

Disfunction of Kv channel function has been associated with neuronal hyperexcitability and implicated in neurodevelopmental and neurodegenerative disorders [116]. In animal models of PD, inactive Kv channels are linked to reduced dopamine release. Mice over expressing mutant α-synuclein showed increased action potential firing of dopaminergic neurons and disrupted dopamine release which was attributed to the impaired Kv channels [117]. KCa3.1 channels are the major K^+^ channels in the ECs and astrocytic endfeet and are shown to be involved in ischemia induced K^+^ fluxes leading to EC swelling and subsequent BBB disruption [118]. A recent study has demonstrated that treatment with KCa3.1 channel inhibitor TRAM-34 significantly reduced brain edema induced by ischemic stroke in rats [118]. The involvement of BBB specific K^+^ channels in neurodegenerative diseases will be discussed in section 5.1.3.

### 4.4. Cl^-^ channels

Cl^-^ channels are expressed abundantly in astrocytes, pericytes and ECs wherein they play important roles in control of membrane potential, cell volume homeostasis and regulation of cellular processes such as proliferation and apoptosis [33, 119, 120]. Cl^-^ transport across the BBB is important to facilitate movement of major cations such as Na^+^, K^+^ and Ca^2+^ and thus plays a critical role in ion regulation [121]. Among the 9 different voltage gated Cl- channels (ClCs) identified in mammalian tissue, ClC-2, -3, -6 and -7 are the major members expressed in the brain [121]. Astrocytes express ClC-2, Ca^2+^ activated Cl^-^ channel bestrophin1 (Best1), maxi Cl- channels (MAC), and volume regulated anion channel (VRAC) (reviewed in [119]). The role of astrocytic Cl^-^ channels in BBB function is not clear. However, ClC-2 is expressed in the astrocytic endfeet surrounding the blood vessels, where it is predicted to regulate Cl^-^ and blood flow. Research indicates that genetic knockout of ClC-2 in mice results in extensive reactive astrogliosis, brain inflammation and increased BBB permeability during the aging process [122]. Capillary pericytes express the Ca^2+^ activated Cl^-^ channel, TMEM16A or anoctamin and several members of voltage dependent ClC family: ClC-2, -3, -4, -6 and -7 [30]. These channels play an important role in regulation of membrane potential by coupling to several Ca^2+^ sources, including IP3 receptor and TRP channels that modulate the vascular function. For example, Ca^2+^ mediated activation of TMEM16A causes membrane depolarization, amplifies capillary pericyte contraction and reduced CBF after ischemia in mice [123]. Pharmacological inhibition of TMEM16A reduced the ischemia-evoked contraction and death of pericytes, improved postischemic CBF, and reduced brain hypoxia and infarct size after ischemia [123]. This study highlighted the potential therapeutic value of TMEM16A inhibition to improve CBF in conditions of impaired microvascular blood flow. TMEM16A is also expressed in ECs and forms Ca^2+^ activated Cl^-^ channels to modulate resting membrane potential and [Ca^2+^]_i_. These changes regulate cell proliferation, migration, control of membrane potential, regulation of cell volume, and smooth muscle contraction. Importantly, its activity also contributes to the regulation of BBB trans-endothelial permeability [124].

The CNS Cl- channels not only regulate neuronal excitability but also indirectly impact neuronal functions by modulating gliotransmitter release from astrocytes, influencing neurological disorders (reviewed extensively by Wang et al., 2023 [125]). Disruption of ClC-2 expression for example is associated with astrocyte activation and progressive neurodegeneration in aging mice [126]. Dysregulated Cl^-^ channels cause excessive neurotransmitter release, leading to neuronal impairment. This dysregulation, particularly during astrocytic swelling, contributes significantly to neurological disorder pathogenesis, impacting tissue damage and secondary neurotoxicity. Cl^-^ channels, like VRAC and Best1, modulate the release of excitatory amino acids, exacerbating neuronal function impairments during acute brain injuries [127]. Furthermore, Cl^-^ channels participate in cell apoptosis via endoplasmic reticulum (ER) stress, a mechanism associated with neurological disorders like AD, PD, and amyotrophic lateral sclerosis (ALS). Blocking chloride channels shows promise in preventing apoptosis through the ER-stress pathway, suggesting therapeutic potential against neurodegenerative diseases [128].

### 4.5. Na^+^/H^+^ exchangers (NHEs)

NHEs are a family of membrane transporter proteins, encoded by the SLC9A gene subfamily and NHE1 is the best characterized member of this family expressed abundantly in the brain [129]. It mediates the exchange of extracellular Na^+^ for intracellular H^+^ (H^+^_i_)and plays an important role in regulating intracellular pH (pH_i_), as well as cellular volume, important for various physiological processes [130]. It is essentially quiescent at normal pHi and can be activated by intracellular acidification, osmotic cell shrinkage, growth factors and hormones, and pathological conditions such as hypoxia and ischemia [131]. In response to these triggers, NHE1 together with the bicarbonate transporters functions to extrude excessive protons from the cell. However, increased NHE1 activity also causes an overload of [Na^+^]_i_ that forces the NCX into reverse mode and increases [Ca^2+^]_i_ to mediate a cascade of signaling events that can have deleterious effects on cell survival [132]. In the BBB, NHEs are mainly expressed in ECs, pericytes, and astrocytes. In ECs, ~80% of NHE1 protein is located in the luminal membranes of ECs and contribute to pHi recovery after intracellular acidification, which can be inhibited by NHE1 specific inhibitor HOE642 [133]. Ischemia stimulates EC NHE1 activity that leads to increased [Na^+^]_i_ and EC swelling contributing to ischemia-induced BBB damage. Recent studies have found that functional NHE1 expression in ECs can be activated in response to high extracellular K^+^, which contributes to breaches in BBB integrity and therefore increased drug uptake [134].

There are some studies that demonstrate the presence of the NHE1 in BBB ECs [134, 135]. However, research on the expression and function of NHEs in pericytes remains limited. In cultured human brain microvascular pericytes, pHi is predominantly regulated by NHE1 [136]. Extracellular acidosis induces NHE1 activity and generates Ca^2+^_i_ oscillation via release of Ca^2+^ from the ER stores suggesting NHE1 operation in reverse mode (H^+^ influx in exchange of Na^+^ extrusion). It has been proposed that cytosolic Ca^2+^ increases mediate the phosphorylation of CaMKII and cAMP responsive element-binding protein (CREB), which initiates gene transcription to regulate a variety of cellular responses in human CNS pericytes [137].

In the astrocytes, NHE1 is the most important ion transporter responsible not only for pHi regulation but also for the maintenance of [Na^+^]_i_ [132]. The steady-state pHi in mouse cortical astrocytes is reported at 7.0-7.2 maintained by NBC and NHE1 activities [138]. Acidic astrocytic pH_i_ stimulates a rapid increase in NHE1 activity disrupts Na^+^ and Ca^2+^ homeostasis, reduces Na^+^ dependent glutamate uptake. These ionic perturbations induce astrocyte swelling and promotes release of glutamate, proinflammatory cytokines such as interleukin (IL)-1β, IL-6, tumor necrosis factor (TNF)-α, and matrix metallopeptidase (MMP)-9 which has a detrimental effect on BBB integrity [139, 140]. Targeted deletion of *Nhe1* gene in GFAP^+^ reactive astrocytes resulted in reduced endothelial transcytosis, swelling of astrocyte end feet and ECs, structural disruptions of TJs and overall BBB impairment following ischemic stroke in mice. This specific loss of *Nhe1* in astrocytes led to reduced astrogliosis, diminished infarct volume, and improved cerebral perfusion post ischemic stroke [139]. Moreover, in a model of chronic cerebral hypoperfusion, which replicates the vascular contributions to cognitive impairment and dementia (VCID), the inhibition of astrocytic NHE1 resulted in the reduction of reactive astrocyte gliosis, preservation of white matter and hippocampal integrity, and enhancement of cognitive function by decreasing ROS production and inflammation [141].

Abnormal NHE1 activation has been linked to various neurological disorders such as epilepsy, dementia, and ischemic stroke [7]. Preclinical studies have shown deleterious effects of sustained NHE1 activation during cerebral ischemia and reperfusion. Inhibition of NHE1 either pharmacologically or via genetic knock down, has shown neuroprotection and aiding in neurological recovery [139, 142]. NHE1 is also known to modulate the electrical properties of neurons potentially triggering seizure activity. Studies in mice with nonfunctional NHE1 gene have shown the development of epileptic seizures, ataxia, and decreased growth [143]. Moreover, increased NHE1 activity in astrocytes triggered by HIV-linked gp-120 and cytokines, potentially leads to elevated pHi and enhanced release of glutamate through reversal of Na^+^-dependent glutamate transporters, contributing to the development of AIDS-related dementia [144].

### 4.6. Na^+^-dependent HCO_3_^-^ transporter 1 (NBCe1)

The NBCe1 protein is expressed in astrocytes in the brain and is the major regulator of intracellular and extracellular pH [145]. NBCe1 mediates the electrogenic transport of Na^+^ and HCO_3_^-^ across plasma membranes and exists in five splice variants, NBCe1 (-A through -E), which are abundantly expressed in the brain [146]. These variants share common traits, including voltage dependence, independent of Na^+^ and Cl^-^, and susceptibility to inhibition by stilbene compounds. In astrocytes, NBCe1 acts with a stoichiometry of 1Na^+^:2HCO_3_^-^ and can operate in an outward mode, extruding Na^+^ and HCO_3_^-^ and causing intracellular acidification [138]. However, reverse mode activation of NBCe1 can occur in astrocytes in response to membrane depolarization, elevated extracellular HCO_3_^-^, and Na^+^ concentrations, leading to HCO_3_^-^ and Na^+^ influx, and astrocyte swelling [147]. A recent study has indicated that NBCe1 in astrocytes is required for its normal morphological complexity and BBB function [148]. Targeted *Nbce1* gene deletion in Aldh1l1^+^ astrocytes has shown to reduce astrocyte processes, and astrogenesis coupled with increased frequency of Ca^2+^ waves in the astrocyte soma [148]. Astrocytic *Nbce1* knockout brains also showed altered vasculature with increased blood vessel diameter and volume. Moreover, the ECs exhibited a 2-fold increase in EC marker expression and a notable upregulation of AQP4 protein expression. Additionally, the blood vessels displayed a deficiency in the coverage of TJ proteins ZO-1 and claudin 5, hinting at a plausible role in in modulating BBB integrity [148]. These findings collectively point towards abnormal vasculature largely due to the deficiency of NBCe1 in astrocytes. However, the specific role of NBCe1 in neurological disorders remains elusive.

### 4.7. Na-K-2Cl cotransporter-1 (NKCC1)

The NKCC1 belongs to the SLC12 family of cation-chloride cotransporters (CCCs), expressed abundantly in the brain. It couples Na^+^, K^+^ and Cl^-^ transport into the cell, with a stoichiometry ratio of 1Na^+^:1K^+^:2Cl^-^ [149] and play important roles in the regulation of intracellular chloride concentration [Cl^-^]_i_. NKCC1 is highly expressed by multiple cell types, including immature neurons, astrocytes, oligodendrocytes, ECs and epithelial cells of the choroid plexus [149]. In all these cells, the activity of NKCC1 regulates [Cl^-^]_i_ to maintain their cellular volume amidst fluctuations in extracellular osmolarity and intracellular solute content. This mechanism is crucial to prevent excessive swelling or shrinkage that could compromise cellular structural integrity. Disruption in NKCC1 expression and activity can alter [Cl^-^]_i_ homeostasis, consequently disrupting cell volume and altering the regulated transport of ions across the blood-brain and epithelial barriers.

NKCC1 is expressed abundantly in BBB ECs where it is predominantly located in the luminal membrane of the ECs and plays an important role in the formation of ionic edema by loading Na^+^ into ECs [16]. Its presence in the ECs of leptomeningeal vasculature at the capillary level is shown to play role in CSF production throughout the CNS [150]. Highest levels of NKCC1 expression have been detected in the ECs of embryonic mouse cortex indicating its role in BBB development [151]. Hypoxia, aglycemia, hyperglycemia and moderate to severe ischemia induces increased expression of NKCC1 in ECs which along with NKA increases secretion of Na^+^ from blood into the brain parenchyma leading to edema [152, 153]. In addition to acting as an ion transporter, NKCC1 also participates in interactions with the actin cytoskeleton [154]. F-actin stress fibers present in injured ECs leads to endothelial contraction and disruption of TJ protein ZO-1, which in turn leads to BBB damage [155]. Studies have shown that NKCC1 knockdown or its inhibition leads to decreased expression of F-actin in ECs highlighting its role in regulating cytoskeleton dynamics [156].

In capillary pericytes, NKCC1 protein expression has been detected in CD13^+^ cells in the perinatal mouse brain indicating its role in the development of BBB [157]. Additionally, NKCC1 mRNA expression has been confirmed in the capillary pericytes of the adult brain cortex. In both pericytes and the smooth muscle cells within the vasculature, there exists a high [Cl^-^]_i_ concentration, maintained by the actions of plasma membrane NKCC1 and the Cl^-^/HCO_3_^-^ exchanger AE2 [158]. Recent studies highlight NKCC1’s significant role in the regulation of pericytes contractility [123]. Inhibition of NKCC1 activity in mouse cortical slices with its potent inhibitor bumetanide have demonstrated the attenuation of endothelin-1 mediated capillary contraction indicating the dependency of capillary pericyte contractility on the transmembrane Cl^-^ gradient established by NKCC1 activity [123].

NKCC1 protein is abundantly expressed in astrocyte cell soma as well as processes and plays a crucial role in maintaining [Na^+^]_i_, [K^+^]_i_ and [Cl^-^]_i_ [157]. Unlike neurons, astrocytes preserve high activity of NKCC1 throughout development and therefore, have higher intracellular [Cl-]_i_ [159]. The NKCC1 function in astrocytes is critical for regulating cell volume and counteracting the astrocytic shrinkage due to osmotic stress [7]. Astrocytic NKCC1 activation is also associated with elevated extracellular K^+^ concentrations, resulting in cellular swelling due to co-transport of water [160]. In adult brain, astrocytic NKCC1 has been implicated in withdrawal of fine astrocytic processes from synapses following long-term potentiation induction, highlighting its role in synaptic modifications associated with a memory trace [161]. Increased NKCC1 expression in astrocytes has been seen in various pathological conditions and overstimulation of NKCC1 leads to astrocyte swelling, which subsequently contributes to BBB damage, cerebral edema, and neurological dysfunctions [7].

Upregulation of NKCC1 is a common feature associated in various neurological conditions such as stroke, hemorrhage, TBI, spinal cord injury and brain tumors. Inhibition of NKCC1 using bumetanide (BMT) has shown protective effects by decreasing inflammatory response, neuronal damage, and edema formation in TBI and spinal cord injury models [162, 163]. Additionally, NKCC1 upregulation in choroid plexus was detected in a rat model of posthemorrhagic hydrocephalus, and intraventricular BMT injection reduced CSF hypersecretion [164]. High expression of NKCC1 is detected in several human gliomas associated with poor prognosis. Inhibition of NKCC1 by BMT reduced glioma cell migration and invasion of peritumor tissue in vivo and promoted apoptosis [165, 166]. In rodent models of HD, upregulation of NKCC1 was observed, and BMT administration rescued altered GABAergic transmission, cognition, and motor deficits [167]. Moreover, BMT attenuated motor deficits in PD mouse models [168]. NKCC1 upregulation has also been reported in HD patients, and BMT ameliorated motor symptoms in PD patients.

### 4.8. Na^+^-Ca^2+^ exchanger (NCX)

NCX is a plasma membrane antiporter, which mainly maintains cytosolic Ca^2+^ homeostasis in cells. All three isoforms of NCX (NCX1—NCX3/SLC8A1—A3) are expressed in adult CNS, which can be detected in heterogeneous cell populations, including neurons, astrocytes and epithelial cell [169]. All NCXs were often identified around blood vessels where perivascular astrocytic endfeet and ECs are located. In astrocytes, NCXs are enriched at distal processes surrounding synapses, with NCX1 transcript being the most predominant [7, 170]. As an electrogenic transporter, NCX regulates Na^+^- and Ca^2+^ dependent signaling, as well as membrane potentials in the brain. It has been identified that NCX operates in forward mode at low [Na^+^]_i_, which mediates influx of 3 Na^+^ and 1 Ca^2+^ extrusion across the cell membrane. However, when Na^+^_i_ is elevated, NCX can operate in the reverse mode which couples Na^+^ efflux to the Ca^2+^ influx [7, 171]. In astrocytes and ECs, NCX is involved in the regulation of Na^+^_i_ and Ca^2+^_i_ dynamics, important for BBB integrity and function. Ca^2+^ induced endothelial cytoskeleton contractions were proposed to prolong BBB rupture following osmotic stress [172]. Pharmacological studies show NCX blockers have a synergistic impact on BBB opening during hypertonic infusions and alter the generation of brain vasogenic edema following radio frequency injuries [170]. [Ca^2+^]_i_ transients in astrocyte endfeet can cause cerebrovascular vasoconstriction, which suggests that NCX activity in astrocytic endfeet may play an important role in the glial control of brain microcirculation [170]. In addition, NCX participates in the activation of resident cells in the brain, and leads to the destruction of the BBB. NCX in astrocytes is proven to be involved in Ca^2+^ induced ROS generation, DNA ladder formation, and nuclear condensation [173]. Ca^2+^ overload in brain microvascular ECs increases brain ROS levels by NADPH oxidase 5 (NOX5), which contributes to BBB breakdown [174].

Changes in the activity of NCX isoforms have been implicated in various CNS pathologies, including stroke, multiple sclerosis, hypoxia ischemic encephalopathy, and AD, suggesting their potential as a pharmacological target [175]. In mice, NCX1 overexpression/activation has demonstrated to reduce ischemic volume and improve neurological deficits following ischemic stroke [176]. Knockdown of NCX3 in mice has been shown to impair oligodendrocyte proliferation, leading to the early onset of experimental autoimmune encephalomyelitis accompanied by worsened clinical symptoms [177]. Exposure of neuronal cultures to Aβ_1-42_ induces calpain mediated cleavage of NCX3 isoform that generates hyper functional form of the antiporter that, by increasing Ca^2+^ content into ER, delays caspase 12 activation and neuronal death [178]. The direct research implicating the role of NCX in BBB regulation is currently limited.

## 5. Role of BBB ion channels and transporters in neurodegenerative disorders

It has been well established that both astrocytes and ECs play vital roles in maintaining the functionality and integrity of the BBB. Astrocytic endfeet contribute significantly to BBB regulation by maintaining ion and water balance. Additionally, soluble factors derived from astrocytes have been found to induce essential aspects of BBB function [179]. ECs on the other hand, facilitate the bidirectional transport across the brain through ion transporters, protein and peptide carriers and active efflux transport. Moreover, TJ proteins between brain ECs also greatly uphold the structure of BBB.

Compelling evidence indicates that following acute brain injuries like ischemic stroke and brain trauma, rapid activation of ion channels and transporters within the ECs and perivascular astrocytes disrupts ion homeostasis and contributes to inflammation, increased BBB permeability and brain damage as previously reviewed [7, 11]. However, the impact of dysregulated ion channels and transporters within BBB-specific cells in neurodegenerative diseases including AD and vascular dementia remains understudied.

### 5.1 Ion channels and ion transporters in BBB dysfunction in AD and VaD

In AD, there is emerging evidence suggesting aberrant activities of ion transporters/channels play a role in glial cell activation causing neuroinflammation and neuronal damage. Moreover, they contribute to impaired perivascular clearance of Aβ and disrupts CBF leading to neurodegeneration [180]. In addition, Aβ fibrils and aggregates deposit in the extracellular spaces of the brain tissue and around the vasculature of the brain, causing cerebral amyloid angiopathy (CAA) which damages the vessel wall leading to BBB permeability, vessel occlusion or rupture [181]. In the context of AD and the BBB, our understanding of ion channel and ion transporter dysfunction primarily stems from the interactions between the BBB cell types and accumulated Aβ plaques [181]. For VaD, dysfunction of ion channels and transporter primarily arises from chronic cerebral hypoperfusion, which impairs the BBB due to alterations in the microenvironment and neuronal toxicity impacting BBB specific cells that express essential ion channels and transporters [182]. The vascular pathology associated with VaD results in chronic cerebral hypoperfusion, reducing the availability of ions from the bloodstream for perivascular cells to meet the demands of the brain parenchyma [182]. Nonetheless, it is important to note that there remains limited literature providing explicit evidence for alterations in ion channel/transporter expression and activities within the perivascular cells of the BBB in AD and VaD brains. Evidence suggests that the disruption of Na^+^, K^+^, Ca^2+^ and Cl^-^ homeostasis particularly in astrocytes and ECs contributes to a proinflammatory cascade that precedes the onset of AD development [180]. The underlying mechanisms include compromised perivascular drainage and impaired BBB transport and vascular dysfunction [183]. A post-mortem study demonstrated significant BBB permeability and evidence of cerebral hypoperfusion in patients with AD, VaD, and mixed AD, this clinical evidence supports the hypothesis that vascular pathology may influence the ionic regulation of BBB specific cells as a compensatory response to reduced CBF [184]. Studies focusing on astrocytes and ECs, at the BBB have discovered homeostatic deficits in ion channels/transporters contributing to AD/VaD pathology. Table 2 presents an overview of key BBB ion channels/transporters and their implication in AD and VaD.

#### 5.1.1. BBB specific NKA dysregulation in AD/VaD

Significant research has firmly established a connection between AD and reduced levels of NKA, resulting in Na^+^ and K^+^ imbalance and loss of Ca^2+^ homeostasis. This ionic dysfunction significantly contributes to vascular dysfunction, inflammation, and neurodegeneration [185-187]. However, despite this understanding, the precise cellular identities involved in these mechanisms remain unclear. Ionic imbalances have been observed in cultured mouse astrocytes, when exposed to Aβ_25-35_ and Aβ_1-40_ leading to a 2-3 fold increased [Na^+^]_i_ and 1.5 fold increase in [K^+^]_i_ [186]. These alterations closely resemble the levels detected in the brain tissues of individuals with AD indicating dysfunctional NKA [186]. The Aβ treated astrocytes also exhibited decreased expression of NKA indicating its depressed activity contributing to ion imbalance [186]. Conversely, a recent study has revealed increased levels of α2 subunit of NKA (α2 -NKA) within astrocytes in postmortem AD human brain tissue [188]. In a mouse model of tauopathy, pharmacological inhibition of α2-NKA suppressed the neuroinflammation and brain atrophy and α2-NKA knockdown prevented the accumulation of tau pathology [188]. Recently, in an in vitro blood cell cultures and ex vivo blood vessels, it has been demonstrated that patient derived Aβ assemblies bind to the α3 subunit of NKA in caveolae on ECs and induces ROS production and activates PKC. The increased PKC stimulates inactive form of endothelial nitric oxide (eNOS) that causes vessel constriction and EC damage [189]. In a recent study, Philbert et al., [190] utilized a plasma mass spectrometry (ICP-MS) to measure the concentrations of Na^+^ and K^+^ in the human post-mortem tissues from VaD cases, revealing increased Na^+^ and reduced K^+^ levels in hippocampal tissue, potentially due to dysfunction of NKA contributing to hippocampal atrophy and cognitive impairment in VaD [190]. These findings raise the possibility that reduced activity of the NKA may underlie the observed alterations in Na^+^ and K^+^ levels. Moreover, in a separate investigation led by Adav et al., [191], an in-depth analysis of the proteomic composition of post-mortem brain samples from VaD patients indicated a substantial increase in NKA protein expression, accompanied by the deamidation of both catalytic and regulatory subunits of the NKA protein. These findings emphasize the central role of NKA in preserving ion homeostasis and raise the possibility that the specific functions of various NKA isoforms in distinct cell types may bear critical implications for the pathophysiology of both AD and VaD.

**Table 2:** Ion channels and transporters within BBB and their dysregulation in AD and VaD.

Ion channel/ transporter	Transported ion	BBB cells	Changes in AD/VaD	Refs
**Na^+^-K^+^-ATPase (NKA)**	Na^+^, K^+^	Endothelial cells (ECs), Astrocytes (As), and Pericytes	Reduced expression and activity in As and ECs in mouse AD models.	[186]
			Reduced activity in VaD brain.	[190]
			Aβ binding to NKA causing vessel constriction in vessels isolated from AD patient brains and EC damage in EC cultures.	[189]
**Kv1**	K^+^	ECs, Smooth muscle cells	No reports.	[107]
**Kv3.4**	K^+^	As	Upregulation in as exposed to Aβ oligomers and in As of Tg2576 AD mice.	[221]
**KCa3.1 and BK channels**	K^+^	ECs, As	Increased activity in As, Reactive gliosis, pro inflammatory cytokine production in SAMP8 mouse model of AD. Decreased EC BK activity, capillary constriction and reduced CBF in APP23 mouse model of AD.	[218], [220].
**Kir2.1**	K^+^	ECs	Kir2.1 overexpression, reduced vasodilation, and neurovascular coupling in APP; 3xTg-AD and 5xFAD mice.	[224], [225]; [226].
**Kir4.1**	K^+^	As	Reduced expression in As in VCID mouse model, Postmortem human AD brains, and in APPSw (Tg2576) and APPSwDI AD mice.	[216],[214]
**Kir6.2**	K^+^	As	Increased expression in reactive as in human postmortem AD samples and in 3xTg-AD mice.	[213]
**CaV1.2**	Ca^2+^	As	Increased expression in reactive As in Aβ PP751 mice.	[195]
**TRPV1**	Ca^2+^	As	Aβ induced increased expression in As, Increase in iNOS, COX2 and TNF-α production.	[227]
**TRPV4**	Ca^2+^	As, ECs	Aβ induced increased activation in As, oxidative stress and astrocyte damage in mouse brain slices; proinflammatory cytokine production.	[205]
**TRPC1**	Ca^2+^	As	Increased activation in As from Tg5469 AD mice, Ca^2+^_i_ overload, inflammation.	[228]
**TRPM2**	Ca^2+^	As, ECs	Increased activation in ECs, Ca^2+^_i_ overload, and EC dysfunction.	[208]
**TRPA1**	Ca^2+^	ECs	Mediates increase in Ca^2+^_i_ in EC line bEnd3 exposed to Aβ42 oligomers. Increased expression within ECs in 5xFAD mice associated with BBB disruption.	[209]
**NHE1**	Na^+^, H^+^	As, ECs	Increased expression in reactive As in BCAS model.	[141]
**NKCC1**	Na^+^, K^+^, Cl^-^	As, ECs	No reports	
**NCX**	Na^+^, Ca^2+^	As, ECs	No reports	
**NBCe1**	Na^+^, HCO_3_^-^	As	No reports	

#### 5.1.2. BBB Ca^2+^ channels/transporter dysregulation in AD/VaD

Most studies investigating ionic dysregulation in AD brains highlights the involvement of astrocytic Ca^2+^ dysfunction [192, 193]. Dysregulated astrocytic Ca^2+^ transporters/channels are the major drivers of neuroinflammation observed in AD. Though specific endfeet Ca^2+^ dynamics and dysfunctional channel or transporters at the BBB in AD brains has not been reported in the literature so far, it is noteworthy that the astrocytic Ca^2+^ signaling constitutes an intricate machinery, well organized in cell soma and fine processes contacting the vasculature orchestrating the BBB pathophysiology. The microdomain Ca^2+^ changes have been observed in the endfeet that are mediated through voltage gated Ca^2+^ channels, TRP channels, SOCE entry or reversed NCX [194]. The Ca^2+^ changes mediated by aberrant activities of these channels/transporters induce gene expression changes, astrocyte metabolism and reactive astrogliosis that can impact the BBB in AD brains [193]. Increased activity of L type voltage gated Ca^2+^ channels has been implicated in pathogenesis of dementia and AD. In a recent study, Daschil et al., has reported that CaV1.2 α1-subunits are highly expressed in reactive astrocytes of 12-month-old transgenic AD mice associated with increased amyloid-β-load [195]. Abnormally large Ca^2+^ signals have been detected in astrocytes isolated from triple transgenic AD mice indicating defects in Ca^2+^ handling [196]. Hyperactivity of [Ca^2+^]_i_ has also been observed in vivo within the plaque associated reactive astrocytes in the brains of animal models of AD [197, 198]. The activation of astroglial purinergic receptors is also implicated in these aberrant Ca^2+^ changes and an increased frequency of astroglial Ca^2+^ oscillations in the pre plaque stage is associated with instability of BBB ECs affecting the vascular tone and local blood flow [199, 200]. Vascular dysfunction is also noted upon exposure of astrocytes to Aβ that increased the frequency of spontaneous Ca^2+^ responses associated with arteriolar lumen diameter instability [199-201]. In these studies, the Ca^2+^ changes were characterized by oscillating cycles of vessel relaxation and constriction [200]. Importantly, the vascular responses corresponded with increased frequency and amplitude of evoked intercellular astrocytic Ca^2+^ waves [197] and was found prominent in the reactive astrocytes close to amyloid plaques [199].

Another path to [Ca^2+^]i overload in astrocytes is shown to be through Aβ-activated TRP channels that include TRPA1, TRPC6, TRPV1 and TRPV4 [114]. This specific path of Ca^2+^ overload leads the astrocytes to express pro-inflammatory factors, activating NF-kB, NFAT and serine/threonine-protein phosphatase 2B (PP2B), inducing an inflammatory phenotype akin to reactivity, which can prove detrimental to the integrity of the BBB [202]. Aβ also mediates activation of astrocytic TRPC1 channels which interacts with Orai1 and stromal interacting molecule, ST1MI that drives the SOCE causing sustained Ca^2+^ signals that can cause inflammation [203]. Additionally, TRPC6 is known to exhibit protective effects on astrocytes including inhibiting astrocyte Ca^2+^ hyperactivity and suppressing the astrocytic inflammatory response [204]. Elevated levels of astrocytic TRPV4 channels in AD brains are implicated in Ca^2+^ mediated astrogliosis, ROS production, and increases in IL-1β, TNF-α, inducible nitric oxide synthase (iNOS), and cyclooxygenase (COX) 2 that induces inflammatory BBB damaging response [205]. Ca^2+^ induced EC dysfunction in AD and VaD is shown to be mediated mainly through the TRP channels [206, 207]. It has also been demonstrated that extracellular Aβ accumulation in AD brains induces oxidative stress in brain ECs, which activates the DNA repair enzyme poly-ADPR polymerase [208]. The increased production of poly-ADPR activates TRPM2 channel, thereby inducing an EC Ca^2+^ overload, EC dysfunction and BBB impairment [208]. In a recent study, Yang et al., [209] reported that exposure of EC line, bEnd3 to Aβ42 oligomer induces Ca^2+^ dysregulation mediated by TRPA1 channels. In addition, their findings indicated elevated TRPA1 expression within ECs in 5xFAD mice, was associated with Aβ accumulation and BBB dysfunction [209].

These studies strongly indicate that Ca^2+^ signaling in astrocytes and ECs is dysregulated within AD brains. Aberrant Ca^2+^ signaling leads to disruption in cellular structures, alter protein expression, increase secretion of pro inflammatory mediators that can impact BBB permeability. Besides TRP channels, dysregulated Ca^2+^ due to abnormal BBB transporter activities discussed in section 4.2 remain unexplored in AD and VaD. Given the important roles that these channels and transporters play in BBB disruption in acute brain injuries, further research is required to understand their potential impact on chronic neurodegenerative diseases like AD.

In the context of VaD, there is a growing interest in Ca^2+^ channels as potential therapeutic targets, although they are not conventionally studied within the specific cell types of the BBB. The hypoperfusion observed in VaD could potentially benefit from Ca^2+^ channel blockers, as they have the capacity to relax smooth muscle cells in the cerebral vasculature. Using BBB permeable Ca^2+^ channel blockers may prove valuable in mitigating excitotoxicity, a phenomenon that leads to neuronal death seen in both VaD [210]. Additionally, recent study by Taylor et al., [211], investigating the CBF component of VaD in a hypertension induced VaD model has shed light on the uncoupling of Ca^2+^ sparked BK Channel mediated vasodilation mechanisms. These mechanisms, which normally promote hyperpolarization and vasodilation, become uncoupled, and leads to the increase in vessel constriction in hypertension models [211]. Given VaD’s reduced blood flow and vascular disease components, it is plausible that downstream effects could influence Ca^2+^ ion concentrations and alter the activities of Ca^2+^ channels and transporters. Therefore, it would be worthwhile to explore the activities of Ca^2+^ channels and transporter in perivascular cells at the BBB in the context of VaD.

#### 5.1.3. BBB K^+^ channels in AD

Abnormal expression of astrocytic KATP and Kir channels is known to contribute to the pathogenesis of AD [212, 213]. Kir6.2 subunits were significantly increased in reactive astrocytes in both patients with AD and in 3xTg-AD mice hippocampus [213]. However, their contribution to BBB pathophysiology is not clear. A postmortem study indicated that reduced Kir4.1 expression in patients with AD exhibited moderate to severe CAA [214]. In mice with varying degrees of CAA, a decrease in Kir4.1 expression was seen in astrocytes at the neurovascular unit indicating its role in loss of BBB integrity and vessel occlusion/rupture [214]. The loss of astrocyte Kir4.1 in AD pathology is similar to the loss of AQP4 at the BBB; both anchor to DP71 dystrophin protein in astrocyte terminal protrusions and play important roles in maintaining BBB integrity [215].

In vascular cognitive impairment and dementia mouse models, Sudduth et al. [216] demonstrated the destruction of astrocyte terminal protrusions and a decrease in DP71, AQP4, and Kir4.1 localization along with neuroinflammation and cognitive dysfunction. AQP4, Kir4.1 and dystrophin 1 were also reduced in autopsied brain tissue from individuals with AD that also display moderate and severe CAA [214]. While reductions in Kir4.1 may occur secondary to changes in the BBB due to increase in Aβ deposition, such reductions in expression may play a role in overall disease progression as well as the development of co-morbidities such as seizures [217] in AD patients. Thus, Kir4.1 may represent a potential therapeutic target in the progression of AD. In addition, increased activity of intermediate-conductance KCa, KCa3.1 in astrocytes is linked to Ca^2+^ overload, astrocyte activation and gliosis [218]. Inhibiting KCa3.1 channel activity with pharmacological agents reduces reactive astrogliosis and production of pro-inflammatory factors, IL-1β and TNF-α [218]. Proinflammatory cytokines can damage the BBB and increase its permeability through activation and destruction of TJs of microvascular ECs [219] and this needs to be further evaluated in the context of AD/VaD conditions. Recently reduced activity of endothelial BK channels was seen in APP23 AD mice that exhibited enhanced cerebral vessel constriction and reduced blood flow to the brain [220] indicated compromised vessel function. Recently, Kv3.4 subunit, mediating fast inactivating K^+^ currents, was shown to be upregulated in reactive astrocytes in both in vitro and in vivo AD models. Selective knockdown of Kv3.4 expression significantly downregulated both reactive astrogliosis and Aβ trimers in the brain of AD transgenic mice [221]. Thus, it is reasonable to predict that changes in K+ channel function may alter K^+^ buffering and signaling at the vasculature leading to further brain impairment.

#### 5.1.4. BBB NHE1 in AD and VaD

NHE1 is specifically found in glial cells of cortical and hippocampal regions of the brain, areas often implicated in AD development and pathology [144]. So far there is no direct evidence to indicate NHE1 mediated BBB impairment in AD or VaD models. However, in an experimental vascular dementia model (BCAS), NHE1 protein is shown to be upregulated in reactive astrocytes and pharmacological inhibition of NHE1 protein with its potent inhibitor HOE642 significantly attenuated astrogliosis and improved hippocampal integrity and cognitive function [141]. The underlying mechanism is that blocking NHE1-mediated H^+^ efflux acidifies astrocytic cytosolic environment, which dampened NOX activity [222]. This led to suppression of NOX-mediated ROS production and pro-inflammatory transcriptome in reactive astrocytes. Considering the well-established role of inflammatory reactive astrocytes in BBB dysfunction [139, 223], it is speculated that increased NHE1 activity in reactive astrocytes in AD and VaD brains could potentially lead to vascular inflammation, contributing to BBB dysfunction associated with cognitive impairment. However further research is necessary to establish a direct correlation between increased NHE1 activity in perivascular astrocytes/ECs and BBB damage in AD or VaD.

## 6. Conclusions and Perspectives

The ion channels and transporters expressed by BBB ECs, astrocytes, and pericytes play key roles in controlling the precise concentrations of Na^+^, K^+^, Ca^2+^, and Cl^-^ across the BBB, providing an optimal medium for neuronal function and synaptic signaling. Dysfunction in these ion channels and transporters leads to osmotic cell swelling, activation of proinflammatory mediators, disruption of TJ proteins, a common phenomenon in acute brain injuries. However, their role in neurodegenerative diseases like AD and VaD, and their contribution to long-term impairments, requires further investigation. Our understanding of how these channels influence disease mechanisms is limited, especially in chronic neurodegenerative conditions. Since ion dysregulation precedes amyloid pathology, irreversible neuronal damage or cognitive decline, detection of dysregulated BBB specific ion channel and transporters could facilitate early identification of neurodegenerative changes. Further research should focus on characterizing these channels in healthy and diseased states, understanding their contribution to BBB integrity or dysfunction. Structure-function studies and exploring drug targets for restoring ion balance in neurological disorders are warranted. Future research directions should explore pharmacological interventions specifically tailored to these BBB channels to restore ionic equilibrium.
